# Reoperative approach after extra-anatomic ascending-to-descending aortic bypass graft

**DOI:** 10.1186/s13019-024-02968-5

**Published:** 2024-07-15

**Authors:** Alice L. Zhou, Deven Patel, Michael P. Robich

**Affiliations:** Division of Cardiac Surgery, Department of Surgery, Johns Hopkins Hospital, 1800 Orleans Street, Zayed Tower, Baltimore, MD 21287 USA

**Keywords:** Extra-anatomic aortic graft, Ascending-to-descending aortic graft, Reoperation, Cardiopulmonary bypass

## Abstract

**Background:**

Extra-anatomic ascending-to-descending aortic bypass grafts have historically been utilized as a safe and effective solution for repairs of complex coarctation of the aorta. However, reports on reoperation in these patients remain rare. We present a case of an aortic valve replacement and coronary artery bypass grafting in a patient with an extra-anatomic ascending-to-descending aortic bypass graft.

**Case presentation:**

The patient is a 59-year-old male with a complex aortic history, including repair of aortic coarctation with an ascending-to-descending aortic bypass graft 13 years prior, was admitted to the hospital for shortness of breath and chest pain that had developed over the past year. On further workup, he was found to have severe bileaflet aortic valve stenosis, non-ST elevation myocardial infarction, and moderate coronary artery disease. He underwent surgical aortic valve replacement and coronary artery bypass grafting. Given his unique anatomy, cardiopulmonary bypass approach involved separate cannulation of the right axillary and left common femoral arteries with cross-clamp of both the aorta and the extra-anatomic graft. Using this approach, the redo operation was successfully performed.

**Conclusions:**

Reports on reoperation after ascending-to-descending aortic bypass grafting are rare. We describe our approach to cardiopulmonary bypass and reoperation in a patient with an extra-anatomic ascending-to-descending aortic bypass graft.

## Background

Extra-anatomic ascending-to-descending aortic bypass grafts have been utilized for repairs of complex coarctation of the aorta with low morbidity and mortality. [1–4] Bypass graft anastomosis sites and anatomic positioning are incredibly important to consider in the event of future redo cardiac operations. Given the inherit rarity of extra-anatomic aortic bypass grafts, reports on reoperation in this clinical setting remain infrequent. Considering the unique and intricate anatomy of these patients, meticulous preoperative planning is crucial when contemplating reoperation, with a special emphasis on the approach to cardiopulmonary bypass (CPB). We present a case of an aortic valve replacement and coronary artery bypass grafting in a patient with a prior extra-anatomic ascending-to-descending aortic bypass graft.

## Case presentation

**Fig. 1:**
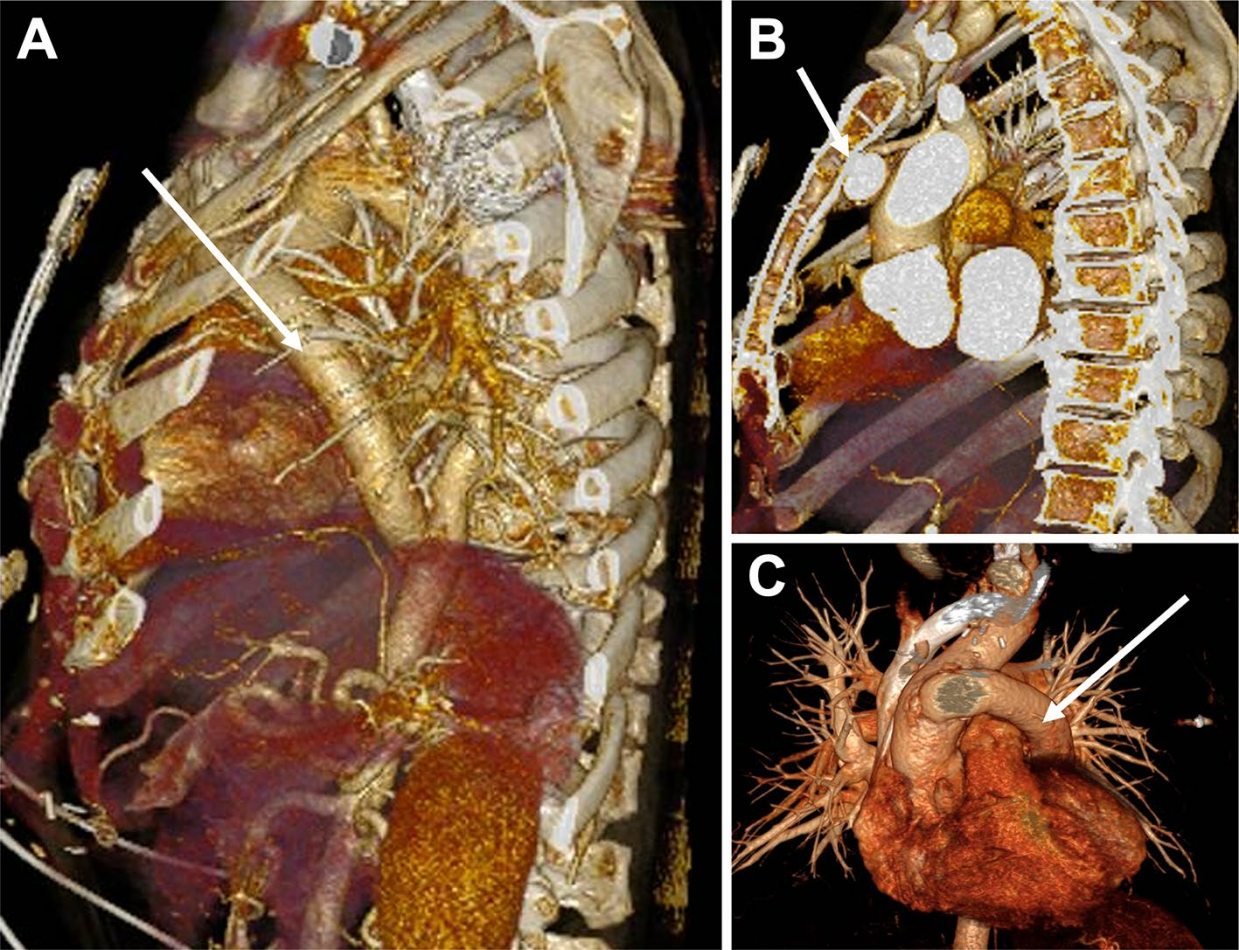
3D reconstruction images of extra-anatomic aortic bypass graft (white arrow). (**A**) Sagittal view demonstrating extra-anatomic aortic bypass graft. (**B**) Sagittal view demonstrating proximity of extra-anatomic aortic bypass graft to sternum. (**C**) Superior view of extra-anatomic aortic bypass graft

The patient is a 59-year-old male with a complex aortic history that included repair of an aortic coarctation with a left subclavian to descending aorta bypass at age 19, complicated by stenosis of the aforementioned graft resulting in recurrent symptomology given his existing aortic coarctation. Accordingly, the patient underwent reoperation with an ascending-to-descending aortic extra-anatomic bypass using a 20 mm Dacron graft at age 45 (Fig. 1). The graft originated at the mid ascending aorta, traversed over the pulmonary artery to the mid descending thoracic aorta. Unfortunately, at age 56, the patient developed massive hemoptysis and was found to have a ruptured large pseudoaneurysm of his original left subclavian descending aorta bypass graft. The patient underwent complex repair of a pseudoaneurysm rupture which included a left common carotid to left subclavian artery bypass, ligation of the intrathoracic left subclavian artery, and occlusion of the proximal descending thoracic aorta using an Amplatzer ™ Vascular plug (St. Jude Medical, Plymouth, MN). 

The patient was admitted to the hospital for shortness of breath and chest pain that had developed over the past year. Upon further workup, he was found to have severe bileaflet aortic valve stenosis and non-ST elevation myocardial infarction (NSTEMI). Transthoracic echocardiogram demonstrated an ejection fraction of 55–60% and bileaflet aortic valve stenosis with a peak gradient of 60 mmHg, mean gradient 33 mmHg, aortic valve area 0.8 cm², and a dimensionless index of 0.26. Chest computed tomography angiography (CTA) demonstrated intact ascending-to-descending aortic bypass with an Amplatzer™ occluder previously described in the proximal descending aorta resulting in total occlusion of the aorta at this level. The aortic root was 44 mm, the ascending aorta was 31 mm, and the aortic area/height ratio was 8.6 cm^2^/m. Left heart catheterization demonstrated obstructive coronary artery disease, with 80–90% luminal narrowing of the proximal left anterior descending and proximal/mid circumflex arteries.

**Fig. 2:**
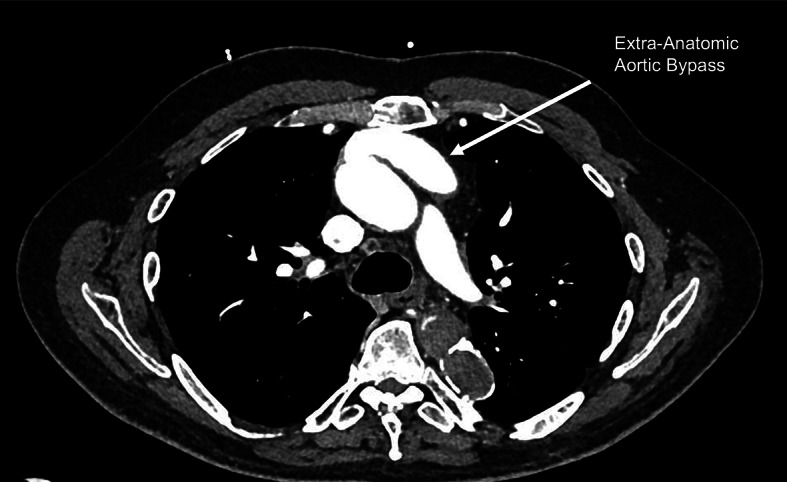
Axial computed tomography image of extra-anatomic aortic bypass sitting behind the posterior sternum

After multi-disciplinary evaluation, plans were made for the patient to undergo redo sternotomy for aortic valve replacement and concomitant coronary artery bypass grafting. Notably, the patient was deemed unsuitable for transcatheter aortic valve replacement (TAVR) due to the significant tortuosity along the path from his only access option, the right carotid artery, and the aortic valve. A significant portion of the preoperative planning centered on devising a safe entry and CPB strategy, taking into account the patient’s prior anatomic vascular reconstruction. This notably involved addressing the occluded proximal descending aorta and the extra-anatomic ascending-to-descending aortic bypass graft, positioned immediately below the posterior table of the sternum (Fig. 2). Preoperative transesophageal echocardiography demonstrated trace aortic insufficiency. Taking into account the aforementioned factors, CPB was initiated prior to sternotomy through separate cannulation of the right axillary artery via an 8 mm Dacron graft and left common femoral artery. A left common femoral venous cannula was also placed with the tip in the superior vena cava. The patient was placed on full CPB with upper and lower body flow. While systemic cooling was carried out to a goal core body temperature of 26 °C, redo median sternotomy was successfully performed with great care to not injure the extra-anatomic aortic bypass graft. The heart did not fibrillate during the cooling process. However, percutaneous defibrillation pads were placed in anticipation for this possibility. Additionally, as soon as we had performed our sternotomy, we carefully dissected out the right superior pulmonary vein and were prepared to place a vent if needed. If the heart did fibrillate prior to completing the redo sternotomy, a left ventricular (LV) vent could have been placed into the LV apex via a small anterolateral thoracotomy incision. The native ascending aorta and extra-anatomical ascending-to-descending aortic graft were both cross-clamped, and the heart was arrested with cold blood cardioplegia administered antegrade. During the operation, antegrade cerebral perfusion was performed and near-infrared spectroscopy (NIRS) monitoring was used to ensure adequate cerebral perfusion.

**Fig. 3:**
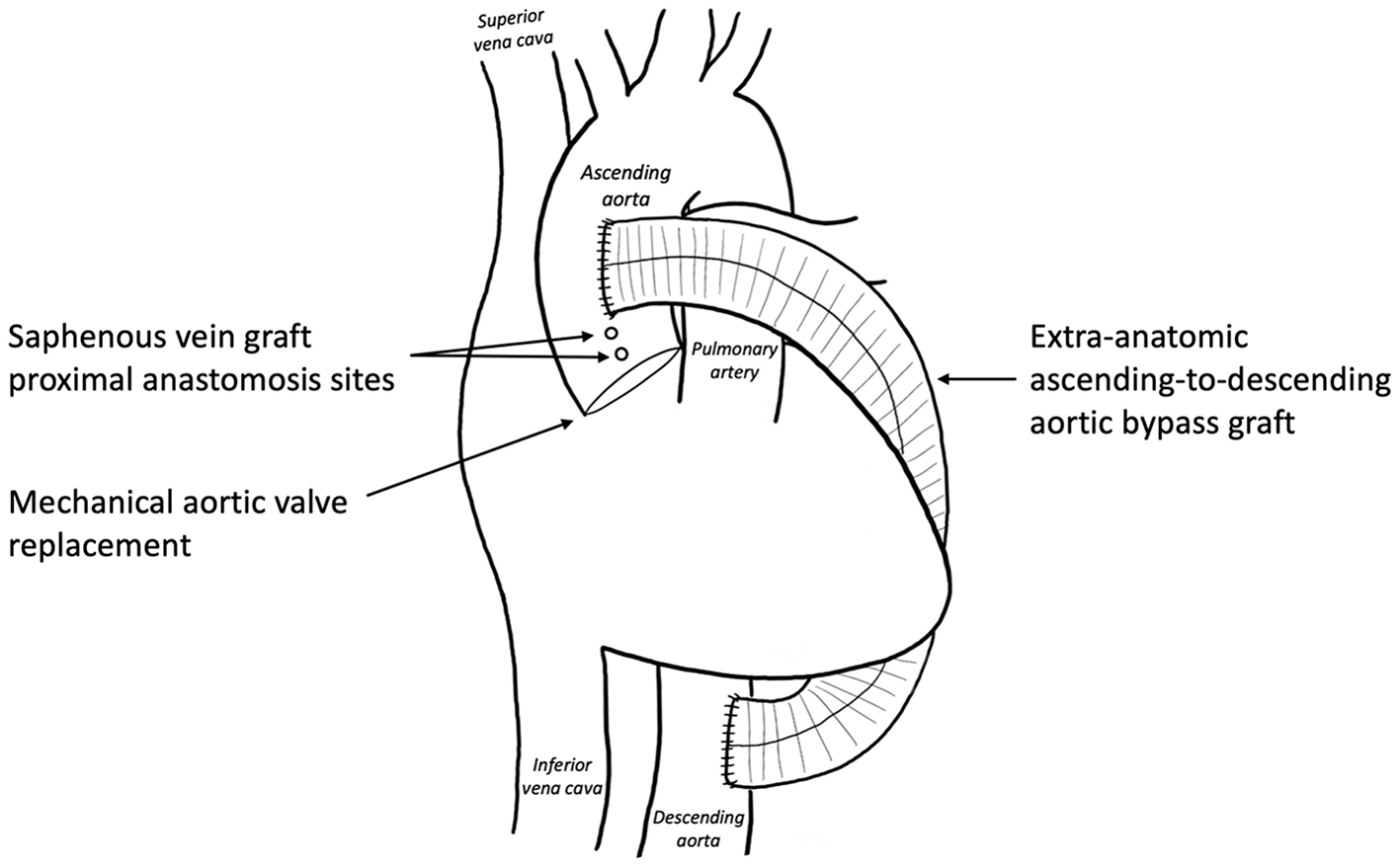
Schematic demonstrating placement of saphenous vein grafts relative to extra-anatomic ascending-to-descending aortic bypass graft

Aortic valve replacement with a 23 mm OnX mechanical valve was performed based on the patient’s age and preferences after a thorough discussion of the risks and benefits of a mechanical versus bioprosthetic valve. Coronary artery bypass grafting x2 was performed on cross clamp, with saphenous vein grafts to the left anterior descending artery and first obtuse marginal artery. The proximal ends of the saphenous vein grafts were anastomosed proximal to the extra-anatomic bypass graft (Fig. 3). Notably, the left internal mammary artery was not utilized as a conduit given the significant dilation of the vessel secondary to the patient’s history for aortic coarctation, as well as the difficulty in mobilizing the vessel in the setting of his extra-anatomical aortic bypass. The patient was then weaned from CPB on moderate inotropic and vasoactive support. Cross-clamp time was 124 min. CPB time was 292 min.

**Fig. 4:**
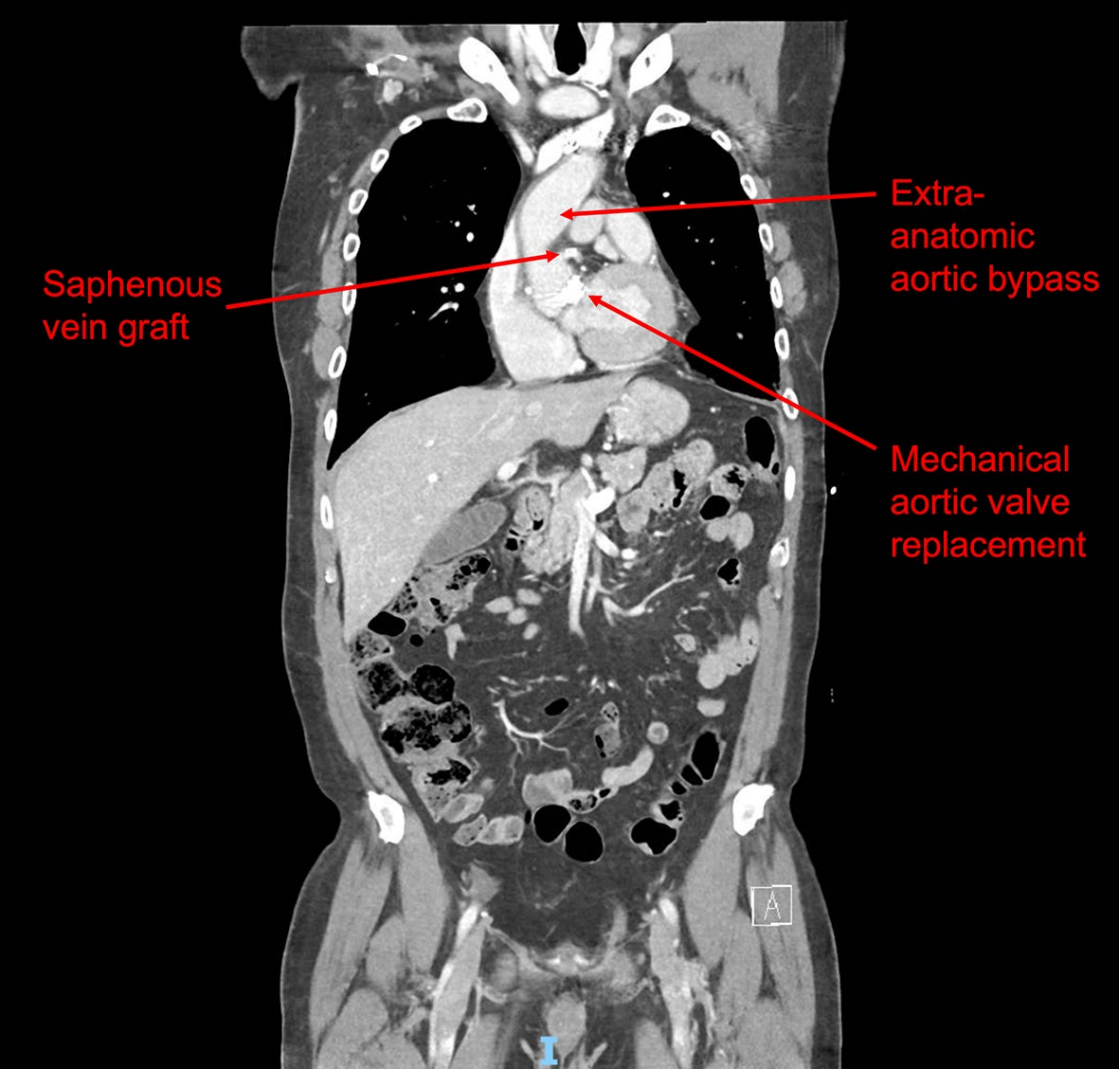
Post-operative coronal computed tomography image of extra-anatomic aortic bypass, saphenous vein grafts, and mechanical aortic valve replacement

His post-operative course was complicated by the need for reintubation on post-operative day 3 for pulmonary edema. He was successfully extubated on post-operative day 7 and discharged on post-operative day 14. He continues to do well 10 months after the operation. Post-operative imaging 8 months after his operation is shown in Fig. 4.

## Discussion and conclusions

For patients with complex aortic coarctation, one option for repair is to perform an extra-anatomic ascending-to-descending aortic bypass. This is a single-stage repair of coarctation that has been shown to have excellent outcomes. [1, 2, 5–8] Multiple approaches to this technique have previously been described, including through a median sternotomy, [9–11] right thoracotomy, [12] and left posterolateral thoracotomy approach. [13] The bypass graft can be configured in different ways, including passing anteriorly or posteriorly to the inferior vena cava along the right side of the heart, or superiorly to the pulmonary artery along the left side of the heart. [1] The positioning of the anastomoses and graft are important to consider for facilitation of future reoperation.

In the current case, the extra-anatomic aortic bypass graft was situated anteriorly, forming end-to-side anastomoses between the proximal ascending and descending aorta. The resulting placement positioned the graft directly beneath the posterior sternal table, rendering a redo sternotomy risky. Consequently, perfusing the upper and lower body separately through the axillary and femoral arteries, respectively, allowed for minimal impact in case the aortic bypass graft needed to be clamped due to an iatrogenic injury during entry. Our case highlights that positioning the graft posteriorly along the inferior vena cava could better facilitate future sternotomy. Furthermore, in this case in which the extra-anatomic bypass graft involved the ascending aorta, by using an upper and lower body cannulation strategy, the aortic bypass graft and native ascending aorta were able to be clamped during CPB to maintain a bloodless field during the aortic valve replacement. In this case, we used a single split arterial line/pump for upper and lower perfusion. In the future, we would consider two separate arterial lines to allow for different flow rates. As previously mentioned, one of the major areas of concern for this case was safe entry into the chest without injuring the extra-anatomic bypass graft. We had accordingly started cooling the patient and prepared all team members for possible circulatory arrest. If an injury were to occur, the patient could have been placed into circulatory arrest, in which case having two separate arterial lines would have enabled for antegrade cerebral perfusion at a lower flow rate (typically 10 cc/kg/minute) while running normal, physiologic flow rates in the lower body given the patient’s lack of continuity within the descending aorta. Further, given the patient’s unique arterial anatomy, the ability to control flow rate to the upper body based on the NIRS would aid in ensuring adequate cerebral oxygenation while avoiding overflowing and inducing cerebral edema. In this patient, a double arterial cannulation strategy was necessary; however, some have advocated for this strategy in patients with normal anatomy for the benefits of shorter cooling and rewarming times, better kidney protection, and improved systemic perfusion stability. [14]

Prior reports on reoperation in patients with ascending-to-descending aortic bypass grafts remain rare. Cabasa et al. described the case of arch reconstruction performed through a trapdoor incision after a previous ascending-to-descending aortic bypass. [15] However, in this case, standard cannulation via the aorta and right atrium was able to be performed. In a case by Rekik et al., urgent reoperation with a Bentall procedure and aortic valve replacement was performed 30 days following the ascending-to-descending aortic bypass grafting due to a pseudoaneurysm at the proximal anastomosis. [16] However, the reoperative approach was not described in detail, and no description of the technique used for placing the patient on cardiopulmonary bypass was presented. Our case provides a framework for successfully placing patients with previous ascending-to-descending aortic bypass grafts on CPB. We describe a safe strategy, with a bailout if there is catastrophic injury to the bypass graft.

In conclusion, we present the case of a redo sternotomy in a patient with an extra-anatomic ascending-to-descending aortic bypass graft. Reports on reoperation and CPB approach in this circumstance remain rare. We describe our surgical approach, which involved separate cannulation of the right axillary and left common femoral arteries and cross-clamp across the extra-anatomic graft. Through this approach, aortic valve replacement and coronary artery bypass grafting were successfully performed.

## Data Availability

No datasets were generated or analysed during the current study.

## Abbreviations

CPB: Cardiopulmonary Bypass
CTA: Computed Tomography Angiography
NSTEMI: Non-St Elevation Myocardial Infarction
TAVR: Transcatheter Aortic Valve Replacement
